# Tetrahydrocannabinol vape-associated cannabis arteritis in a patient with minimal tobacco exposure

**DOI:** 10.1016/j.jvscit.2024.101673

**Published:** 2024-11-08

**Authors:** Morgan Colling, Yousef Souri, Thomas Reifsnyder

**Affiliations:** a Johns Hopkins University School of Medicine, Baltimore, MD; b Division of Vascular Surgery, Department of Surgery, Johns Hopkins Bayview Medical Center, Baltimore, MD

**Keywords:** Cannabis arteritis, Thromboangiitis obliterans (TAO), Delta-9-tetrahydrocannabinol (THC), Vaping, Vasculitis, Claudication

## Abstract

Thromboangiitis obliterans (TAO) is an inflammatory vasculopathy that often presents in young men with substantial tobacco use. Cannabis arteritis is the cannabis-associated counterpart, but there remains controversy over its classification due to overwhelming concurrent tobacco use. A 31-year-old man developed lifestyle-limiting claudication that coincided with vaping high-potency tetrahydrocannabinol. Notably, his tobacco exposure was limited to a remote history of <1 pack-year. His claudication considerably improved after 4 weeks of cannabis cessation. This case demonstrates a rare instance of cannabis arteritis without concurrent tobacco use, suggesting cannabis may act as an independent causative agent of a distinct thromboangiitis obliterans-like arteritis.

Cannabis arteritis is a thromboangiitis obliterans-like (TAO) vasculitis linked to cannabis use rather than tobacco. This non-atherosclerotic vasculopathy manifests clinically as segmental occlusive lesions and obliterations of distal small and medium-sized arteries.^1^^,^^2^ It characteristically affects young adults in their 20s and 30s with a strong male predominance.^2^ Cannabis arteritis is a diagnosis of exclusion in young patients with arteriopathy and significant cannabis use.^3^ It remains challenging to definitively classify and characterize cannabis arteritis within the context of TAO and establish a causal link due to the substantial overlap in cannabis and tobacco exposure in many cases. Although the current evidence suggests that cannabis acts as an aggravating factor, there is not enough convincing data to suggest that it is a primary causative factor with a unique presentation.^2^^,^^4^^,^^5^ This case of cannabis arteritis occurred in an otherwise healthy young man with chronic cannabis usage and tetrahydrocannabinol (THC) vaping but minimal tobacco exposure. He presented with severe bilateral lower extremity claudication, which substantially improved with cannabis cessation. The patient consented to the publication of the details and images included in this report.

## Case description

A previously healthy 31-year-old man presented to the vascular surgery clinic with bilateral lower extremity claudication, right worse than left. He reported a 3-year history of gradually worsening calf tightness that limited his activity to 5 to 7 minutes of walking before needing to rest. Before symptom onset, the patient lived an active lifestyle, playing soccer and rock climbing regularly. The patient exhibited no Raynaud’s symptoms, phlebitis, or upper extremity complaints.

His social history is significant for a 12-year duration of chronic cannabis usage to alleviate anxiety. Notably, his claudication symptoms coincided with the transition from smoking cannabis daily from a pipe to vaping high-potency hash oil several times a day. Other substance use history includes occasional social alcohol use, smoking 2 to 3 cigarettes/day for 1 year in college, and no illicit drug use.

**Fig 1:**
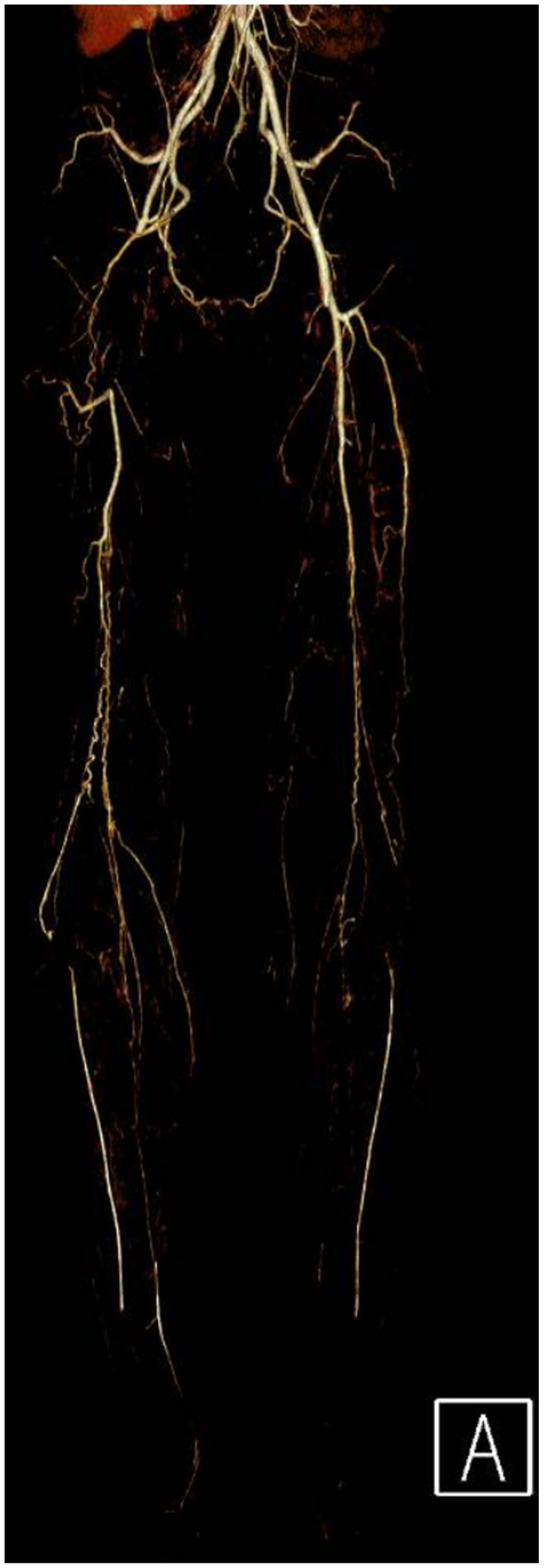
Three-dimensional reconstruction of the patient’s computed tomography angiography with bilateral lower extremity runoff. Note the right superficial femoral artery occlusion with distal reconstitution of the anterior and posterior tibial arteries.

**Fig 2:**
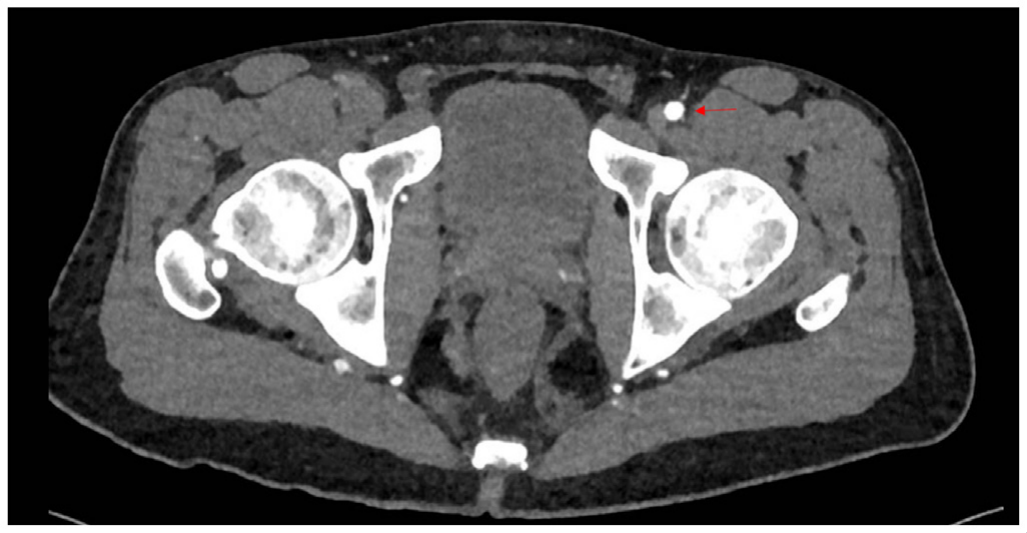
Computed tomography angiography demonstrating occlusion of the right and a normal left common femoral artery (*red arrow*).

Clinical examination revealed non-palpable dorsalis pedis and posterior tibial pulses bilaterally. The lower extremities were warm, without sensory or motor deficits or evidence of tissue loss. Vascular studies demonstrated an ankle-brachial index of 0.36 on the right and 0.45 on the left. Subsequent workup including a computed tomographic angiography, and arterial duplex imaging revealed occlusion of the right common femoral and distal right superficial femoral arteries with reconstitution of tibial vessels. (Figs 1 and 2). The left lower extremity demonstrated a similar pattern of disease without the common femoral artery occlusion. Extensive workup of other possible etiologies for peripheral vascular disease, including hereditary thrombophilias and other vasculitic syndromes, was negative. Labs were notable for normal immunologic and inflammatory markers, a hemoglobin A1C of 5.7, and a normal lipid panel.

The patient received diet and exercise counseling and began taking low-dose aspirin and atorvastatin. He stopped using cannabis and started taking sertraline to address his anxiety. After 4 weeks of these interventions, he was able to exercise 6 days a week, walking for 30-minute intervals before claudicating. Repeat vascular studies at 4 months demonstrated an ankle-brachial index of 0.33 on the right and 0.45 on the left.

## Discussion

TAO is a nonatherosclerotic inflammatory disease of distal small and medium-sized blood vessels, often presenting in young men with substantial tobacco use.^6^ Cannabis arteritis is a contested disease entity that shares many of the same clinical features as TAO, although its presentation is likely confounded by concomitant tobacco exposure in almost all cases. These segmental lesions tend to present in the distal lower extremities and may be associated with distinct proximal lesions.^2^ Although limited by the paucity of arterial biopsy data, the proposed pathophysiology of cannabis arteritis broadly relates to vasoconstriction and direct endothelial damage caused by delta-9-THC, the main psychoactive component of cannabis.^7^, ^8^, ^9^ Although delta-9-THC remains the focus of research efforts, it is also important to consider the toxicity of the products of combustion not only of the tobacco or cannabis, but also of the delivery method such as rolling papers.^4^^,^^10^

It is unclear if cannabis arteritis represents a subtype of TAO or a distinct clinical entity due to the predominance of concurrent tobacco and cannabis use in as many as 97% of cases reported in the literature.^2^ Furthermore, many recorded substance use histories lack the detail necessary to identify other contributing sources of tobacco exposure, such as the cured tobacco leaves used in blunt wraps.^4^ This case represents a rare instance in which there is minimal exposure to tobacco in both smoking history and “cross-contamination” of cannabis. This patient presented with extensive proximal lesions to the common and superficial femoral arteries and claudication without ischemic ulcerations, which is quite unusual for TAO and may better characterize the unique features of cannabis arteritis.^6^^,^^11^ To the authors’ knowledge, this also appears to be one of the first case reports linking THC vaping to cannabis arteritis.

This patient reports a 12-year history of habitual cannabis use, mainly by a water pipe or a dry pipe. In both cases, the user inhales delta-9-THC produced from combustion of the cannabis flower.^12^ The patient denies mixing in tobacco products or using blunt wraps or rolling papers while smoking cannabis. Interestingly, the onset of symptoms began only in the most recent 2 to 3 years after the patient switched to vaping hash oil as the sole method of cannabis consumption. This method uses a heated coil to aerosolize highly concentrated delta-9-THC dissolved in liquid solvents such as propylene glycol and vegetable glycerin.^13^ The potency of cannabis concentrates such as hash oil averages 50% but can soar up to 90% delta-9-THC compared with the 15% potency of traditional cannabis smoking.^12^ This history suggests a dose-response relationship between delta-9-THC and cannabis arteritis without confounding exposure to tobacco or related combustion products, although exposure to additional products of vaporization should be taken into consideration. The scarcity of case reports of TAO-like arteritis with isolated cannabis use likely reflects a commonly occurring co-addiction in the population and, potentially, an additive or synergistic effect caused by concurrent cannabis and tobacco use.^14^

The mainstay of treatment for TAO is smoking cessation, which is difficult for even the most compliant patients. The disease remains quiescent if patients can abstain from tobacco use.^6^^,^^11^ This patient with no recent tobacco exposure experienced marked symptomatic improvement with cannabis cessation along with the initiation of aspirin, a statin, and exercise. Notably, noninvasive testing at 4-month follow-up was unchanged, suggesting that the lesions in cannabis arteritis may primarily be inflammatory as opposed to vasospastic.

## Conclusion

This case highlights the need for more detailed substance use histories to parse out relevant exposures as well as further exploration of the biological mechanisms involved in the pathophysiology of cannabis arteritis. Although there are still many unanswered questions surrounding cannabis arteritis, this case supports the hypothesis that delta-9-THC is an agent capable of causing a distinct TAO-like arteritis independent of tobacco exposure.

## Disclosures

None.
